# Strengthening resilience in the aftermath of adversity: evaluation of the group-based intervention Mind-Spring, a naturalistic mixed-methods study among refugee groups in the Netherlands

**DOI:** 10.3389/fpsyg.2025.1652228

**Published:** 2025-10-23

**Authors:** Elizabeth Nolan, Niels van der Aa, Simon Groen, Simone de la Rie

**Affiliations:** ^1^ARQ Centrum’45, Diemen, Netherlands; ^2^The Academic Center for Dentistry in Amsterdam, Amsterdam, Netherlands; ^3^De Evenaar, Center for Transcultural Psychiatry, GGZ Drenthe, Beilen, Netherlands; ^4^Department of Clinical Psychology, Open University, Heerlen, Netherlands

**Keywords:** refugees, resilience, mental health, well-being, PTSD, prevention, intervention

## Abstract

**Background:**

Refugees often experience multiple traumatic events before and during forced migration, compounded by daily stressors in resettlement, which can erode resilience and increase the risk of serious mental disorders. Strengthening resilience in the aftermath of adversity may help prevent such outcomes. Mind-Spring (MS) is a low-threshold, group-based psychosocial intervention designed to enhance resilience and well-being. Intervention groups were organized by language, cultural background, and gender, and co-facilitated by a mental health professional and a peer educator with lived refugee experience, shared culture and language, and long-term settlement in the host country. This study assessed the feasibility of MS and its impact on four mental health domains among refugees and asylum seekers in the Netherlands presenting with early trauma-related symptoms.

**Methods:**

A convergent parallel mixed-methods study involved four MS groups with 37 participants (72.37% female; mean age 47.4 years, *SD* = 13.6). Feasibility was evaluated through attendance rates and thematic analysis of pre- and post-intervention interviews. Quantitative outcomes were measured using the Brief Resilience Scale-6 (resilience), WHO-5 Well-being Index (well-being), Cantril Ladder (life satisfaction), and Sense of Coherence-Kinderen (sense of coherence). Changes were analyzed using paired *t*-tests, reliable change indices, and multilevel modeling.

**Results:**

High attendance and positive feedback indicated MS is both feasible and acceptable. Participants valued peer support, native-language delivery (Arabic, Dari, Tigrinya, Ukrainian), and the role of peer educators in fostering trust, engagement, and cultural relevance. The intervention’s adaptability and its potential to identify individuals needing additional care underscore its role as both a preventive and bridging approach. Topics considered most helpful included psychoeducation, coping with stress and emotions, and navigating cultural identity. Quantitative analyses revealed medium-to-large effects across domains, with particularly large gains in well-being (*d* = 1.22), and 62.5% demonstrating positive reliable change. Life satisfaction improved progressively during the program.

**Conclusion:**

MS is a feasible, acceptable, and culturally meaningful intervention for resettling refugees, associated with improvements in resilience, well-being, life satisfaction, and sense of coherence. Despite limitations related to sample size and demographic skew, findings add to the evidence base for culturally adapted psychosocial interventions, supporting MS as a promising component within broader refugee integration and mental health services.

## 1 Introduction

Mid-2024, the United Nations High Commissioner for Refugees estimated that there were 43.7 million^1^ refugees, and 8 million asylum seekers worldwide. The number of refugees has tripled over the last decade and is likely to grow due to climate change impacts and global political instability (United Nations High Commissioner for Refugees, 2024). Refugees are often exposed to a cumulative burden of traumatic events before and during forced migration, as well as daily stressors in their country of resettlement. This challenges their resilience, hampering the ability to bounce back or recover from adversity (Smith et al., 2008). Following this definition, resilience can be seen as a dynamic process in which a person responds to negative life experiences. This process can be particularly strained after prolonged and repeated hardship which increases the risk of developing serious mental disorders (Giacco et al., 2017; Gleeson et al., 2020; Steel et al., 2009).

Prevalence rate estimates for trauma-related mental health conditions among this group are approximately nine times higher than those in the general population (Koenen et al., 2017; Moreno-Küstner et al., 2018; Patanè et al., 2022; World Health Organization, 2017). Consequently, there is a large and growing need for adequate mental health interventions for this population. However, in high income countries, despite the mental health needs, the use of mental health services among refugees is limited (Due et al., 2020; Slewa-Younan et al., 2017). Underutilisation may be explained by culturally specific barriers such as cultural taboo, stigma and mistrust, and access barriers, e.g., language problems, complex care systems or waiting lists (Forray et al., 2024; Fuhr et al., 2020; Satinsky et al., 2019).

Strengthening resilience in the aftermath of adversity may prevent the onset or worsening of mental health problems among refugees (Sijbrandij et al., 2017). In high-income countries, group-based interventions are considered important early-access options for this population (Kirsch et al., 2024). In this light, psychosocial resilience-based interventions are increasingly suggested to made routinely available, as part of the healthcare of refugees and asylum seekers (Turrini et al., 2019; Walther et al., 2021). These are largely aimed at preventing mental health problems and promoting well-being. They also address post-migration problems thus making it easier to psychologically adapt to new, stressful environments.

While several psychosocial interventions have shown promise for refugee populations, the evidence base reveals mixed results and methodological variability. The WHO’s Self-Help Plus (SH+) demonstrated effectiveness in preventing mental disorders among Syrian refugees in Turkey but showed diminishing effects at 6-month follow-up in European settings (Acarturk et al., 2022a; Purgato et al., 2021). Problem Management Plus (PM+) has shown more sustained effects, with benefits maintained at 1-year follow-up among Syrian refugees in the Netherlands, though effect sizes were moderate (de Graaff et al., 2024). Importantly, methodological quality across refugee intervention studies varies considerably, with many studies characterized by small sample sizes, limited follow-up periods, or less robust research designs (Nosè et al., 2017; Turrini et al., 2019). This variability in both outcomes and methodology underscores the need for diverse, culturally adapted interventions that can address the heterogeneous needs of refugee populations.

While findings are mixed, they may be shaped by contextual factors that influence both mental health outcomes and the feasibility of implementing interventions. In particular, policy-driven disruptions can heighten stress levels and hinder sustained engagement, thereby reducing the effectiveness of interventions (Figueiredo, 2024). Insecure immigration policies and asylum delays exacerbate stress, insecurity, and trauma among refugees (Laban, 2010; Pluck et al., 2022; Shahzad et al., 2025; Walther et al., 2021). Levels of community engagement can also affect mental health, due to variations in language proficiency, cultural distance, and perceived discrimination (Coumans and Wark, 2024). Restrictions in asylum centers and social isolation further impact coping and emotional states, thereby influencing participation in structured activities such as interventions (Gaeta et al., 2021; Walther et al., 2020; Walther et al., 2021). These factors shape opportunities for meaningful interaction, supportive relationships, and social networks—key to mental health and intervention outcomes (Biddle et al., 2025; Carlsson et al., 2006; Nickerson et al., 2019; van Sint Fiet et al., 2022). Contextual factors may also mediate the feasibility of interventions. Policy instability and crisis-driven emergency responses—such as the sudden influx of Ukrainian refugees—pose substantial challenges to planning and implementation. Especially in low-income countries, service providers and local reception agencies may face constraints in financial resources and staffing, limiting their capacity to consistently deliver interventions. Practical environmental conditions affecting refugees, including housing quality, working hours, and childcare responsibilities, further mediate feasibility (Figueiredo et al., 2022).

Methodological differences among the studies of interventions for refugees mean that the robustness of their findings is not equivalent. Other interventions have been examined in feasibility studies. Nonetheless, several of these interventions have shown promising results. One long-standing psychosocial group intervention for refugees is Mind-Spring (MS), which in 2024 was recognized by the European Commission as a “best and promising practice” for public health promotion (EU, 2024). Developed in the Netherlands in 2004, MS has since been implemented in Belgium, Denmark, Germany, and the United Kingdom. The intervention is delivered in participants’ mother tongue or second language, and targets various cultural and age groups. It consists of eight, 2-hour weekly sessions in groups of 10 to 15 participants. MS is culturally tailored, adapting both content and form to the unique needs and challenges faced by refugees (Uitterhaegen, 2005). Table 1 presents an overview of MS in the Netherlands.

**Table 1:** Brief overview of the Mind-Spring sessions.

Week	Session	Topics	Example exercise
1	Normal (psychological) reactions to abnormal circumstances	Getting to know each other Understanding the aims of MS Establishing general Group Ground Rules Psychoeducation and normalizing stress responses	Scale for preserving a healthy balance between stressful situations and difficulties vs. pleasant situations and activities Relaxation exercise
2	Stress and stress-related complaints	The protective aspect of stress And the difference between healthy and unhealthy stress	How do I recognize stress in my own body?
3	Coping methods for stress	Thoughts and behaviors affect feelings and stress and stress responses can be influenced and managed (Un)healthy coping mechanisms	Vicious cycle of stress Identification of important sources of strength to manage stress
4	Thoughts affect stress	Critical thinking skills regarding one’s own perceptions and beliefs	Reframing thoughts Helpful thoughts
5	Loss and grief	Normal responses in the grieving process	Sharing of own experiences with loss and grief
			Discussing (cultural) rituals supporting the grieving process
6	(Changing) identity	Acculturation process and strategies	Circles of Identity: *Present, Past, Future-me*
7	Cultural differences	Individualistic vs. collectivist cultures	Comparing cultural norms and values between the ‘*I’* and the ‘*We-*culture’
		Parenting in a new cultural setting	Discussing sensitive subjects, e.g., domestic violence and child abuse
8	Reflections and closing ceremony	Taking the leap into the future by reflecting and saying goodbye	Closing ritual Certificates

The intervention aims to foster resilience and mental well-being (Husby et al., 2020). MS is theoretically grounded in both resilience and acculturation frameworks. Resilience is defined here as a dynamic and culturally embedded process, whereby individuals adapt through reciprocal interaction with their environments (Masten, 2019; Ungar et al., 2012). This ecological stance is reflected in session activities that normalize stress reactions, encourage sharing of migration narratives, and foster skills for problem-solving in the host context. Acculturation theory (Berry, 1997) further shapes the curriculum by emphasizing balanced integration—valuing heritage while learning host cultural norms. This approach consistently predicts the highest levels of resilience and positive psychological adjustment among refugee populations (Giles et al., 2025; Han et al., 2016; Wu et al., 2018).

MS has several merits. Firstly, MS can help overcome potential barriers to professional help (Perplies et al., 2021; Uitterhaegen, 2005). In the Netherlands, MS is delivered by two formally trained facilitators: a peer educator with first-hand experience as a refugee and a deep understanding of both Dutch and the participants’ cultural and linguistic backgrounds, and a mental health professional (e.g., a social worker). A registered interpreter is also present at each session to facilitate two-way communication. Together, the team is equipped to refer participants to more intensive mental health care when needed.

Secondly, grouping participants based on their spoken language, gender and cultural background can enhance intervention specificity, given the cultural variability in coping styles and mental health conceptualizations.

Furthermore, the group format is an important aspect, it provides: (i) a low-threshold environment, (ii) a platform to expand one’s social network via contact and exercises with group-members, and (iii) a safe space for problems arising within group dynamics to be addressed (Uitterhaegen, 2005).

Additionally, MS has multiple objectives which are not limited to persons with existing mild psychological complaints. It also aims to prevent the onset or worsening of complaints, by reducing the impact of experienced adverse events *(secondary prevention).*

Additionally, MS fosters empowerment and optimism, e.g., via the peer educator who serves as a role model.

Lastly, MS is a long-standing intervention that has been well received for decades. As the intervention is co-created with asylum seekers and refugees in the Netherlands, its content and delivery are aligned with the participants’ cultural meanings and values (Mahon, 2022).

To date, MS’s potential has primarily been explored via observational studies. Emerging evaluations support MS’s preventive potential. In Denmark, a mixed-methods study of 92 Arabic-speaking male refugees found improvements in well-being and coping, with participants emphasizing stress management and social connectedness (Husby et al., 2020). Perplies et al. (2021) conducted a qualitative process evaluation of MS’s pilot implementation in Germany, identifying facilitators such as personalized outreach, native language communication, and accessible local venues. Barriers included stigma, mistrust of facilitators or peers. This study further highlighted MS’s role in lowering language, cultural, and stigma-related barriers to mental health care. Earlier observational evidence from the Netherlands noted MS’s accessibility, empowerment, and peer role-modeling (Uitterhaegen, 2005). Although no controlled trials of MS have been conducted yet, these findings suggest MS may promote resilience, facilitate integration, and offer a culturally sensitive, low-threshold mental health intervention for refugees. The current study is the first to evaluate multiple mental health related domains among both male and female MS participants from several language groups, i.e., Arabic, Dari, Tigrinya and Ukrainian.

The aim of the current study was to examine the feasibility of MS and evaluate changes in several mental health domains over time. The research questions were: (i) Is MS a feasible intervention that is acceptable, and does it respond to the participants’ needs? (ii) Do resilience, well-being, life satisfaction, and sense of coherence improve during MS? (iii) Do levels of life satisfaction improve throughout the intervention?

## 2 Materials and methods

### 2.1 Design

**Figure 1:**
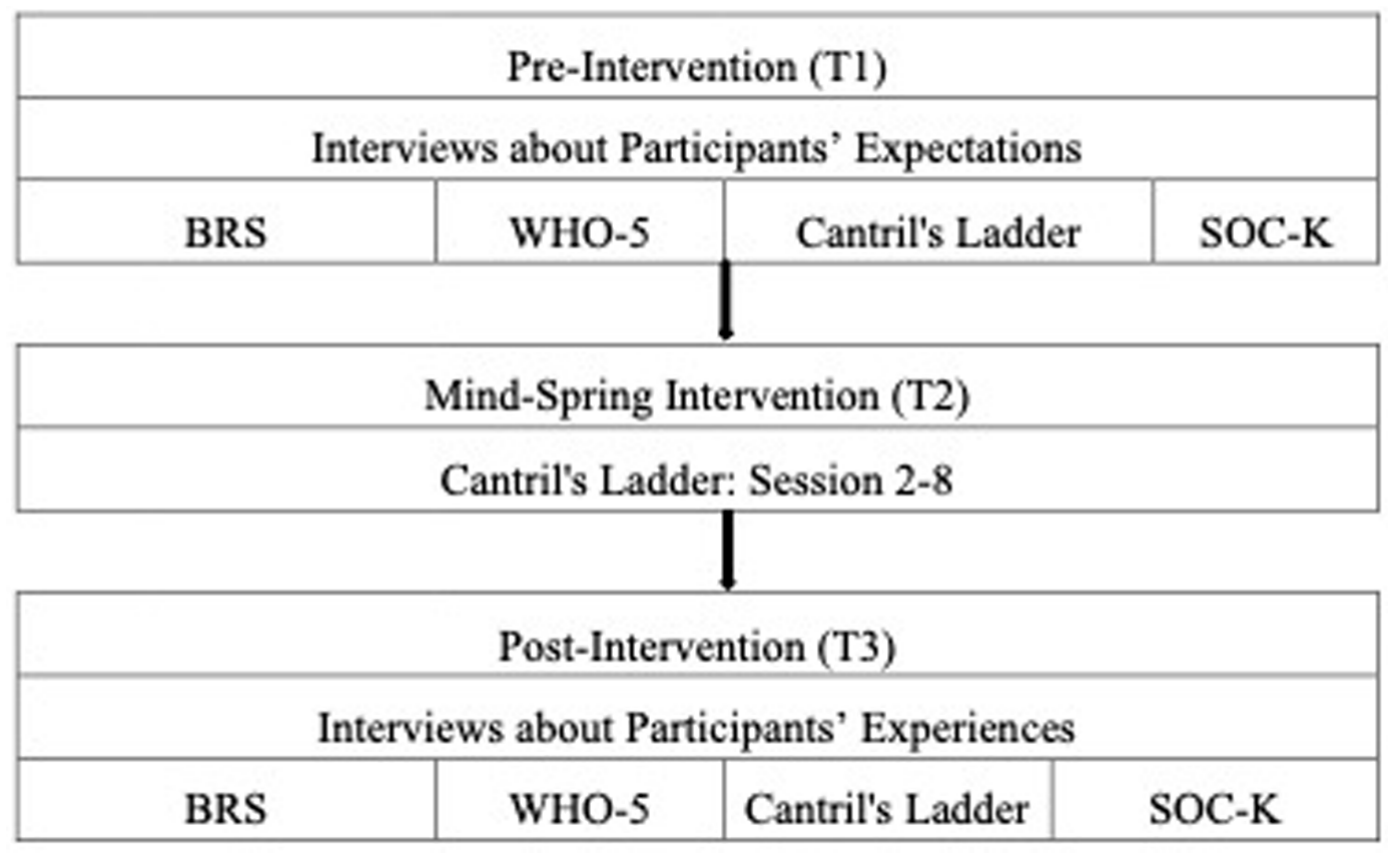
Design of the Mind-Spring evaluation and its measures. BRS, Brief Resilience Scale-6; WHO-5, World Health Organization-Five Well-Being Index; Cantril’s Ladder, The Cantril Ladder of Life, and SOC-K, Sense of Coherence Kinderen.

We used a convergent parallel mixed-methods design (Figure 1), collecting both qualitative and quantitative data. This design allows the quantitative results to be contextualized within participants’ experiences (Orsmond and Cohn, 2015). To enhance rigor, we applied several strategies, including independent coding by two authors, consensus discussions, and triangulation with the quantitative data. Together, these methods strengthen the reliability of the findings and provide a more comprehensive understanding of participants’ experiences and perceived changes through MS.

We assessed MS’s feasibility through intervention attendance and qualitative interviews about participants’ expectations and experiences with MS. Furthermore we measured quantitative changes from pre- to post-intervention using four questionnaires assessing resilience, well-being, life satisfaction, and sense of coherence. Additionally, life satisfaction was measured repeatedly throughout the intervention. No control group was included in the study.

### 2.2 Participants

Participants were refugees and asylum seekers taking part in the MS intervention. Participants were grouped by spoken language and cultural background. The intervention was delivered by a peer educator who has first-hand experience as a refugee and an understanding of the participants’ cultural and linguistic backgrounds, in collaboration with a mental health professional (e.g., a social worker). The initial sample was made up of four intervention groups; an Arabic-speaking men’s group (*n* = 13), a Dari-speaking women’s group (*n* = 15); a Tigrinya-speaking women’s group (*n* = 13) and a Ukrainian-speaking women’s group (*n* = 10). However, as participation in the research project was voluntary, not all of the intervention participants engaged in the study.

Of the *N* = 47 participants who initially consented to the study, *N* = 37 (78.72%) ultimately completed a minimum of two paired questionnaires and/or the repeated measures. No statistically significant differences were observed in pre-intervention scores between participants who withdrew from the study (*n* = 10) and those who completed it. Table 2 presents the sociodemographic characteristics at baseline of the *n* = 37 participants included. Even though only a small number of participants did not provide data post-intervention, there were missing data at each time point for several reasons. Two individuals did not show up at the start of the intervention and three dropped out mid-intervention; one was allocated residence elsewhere and two Ukrainian-speaking participants found work during intervention hours. Also, participants’ absence during some of the sessions resulted in missing data regarding the repeated measures. Further, some participants were reluctant to fill out questionnaires at certain time points. The Tigrinya-speaking participants did not fill out the Brief Resilience Scale-6 (BRS) and Sense of Coherence-Kinderen (SOC-K) at pre and post-intervention, and neither Cantril’s Ladder throughout the intervention sessions. The Arabic-speaking group only filled out Cantril’s Ladder during session 6–8. In addition to practical or logistical challenges, missing data could possibly be due to mistrust. Among refugee participants, mistrust may have stemmed from prior experiences, such as negative encounters with authorities or institutions, or concerns about confidentiality. These factors could have influenced their decision not to continue in the study.

**Table 2:** Sociodemographic characteristics of participants at baseline.

Characteristics		Final sample (*N* = 37)	
		*n*	%
Gender	Male	10	27.6
	Female	27	72.4
Age	20–30	3	8.1
	30–40	9	24.3
	40–50	11	29.7
	50 <	14	37.8
Country of origin	Afghanistan	12	32.4
	Eritrea	6	16.2
	Iran	1	2.7
	Iraq	5	13.5
	Syria	5	13.5
	Ukraine	8	21.6
Juridical status	Dutch citizenship	21	56.8
	Refugee or asylum seeker	16	43.2
Time since arrival in the Netherlands in years	> 1	16	43.2
	1–3	3	8.1
	3–5	6	16.2
	5–10	6	16.2
	10 <	6	16.2

N, Number of participants.

### 2.3 Procedure

ARQ Centrum’45, a specialized center for diagnostics and treatment of psychological trauma, coordinates the implementation of MS in the Netherlands. MS groups are organized in collaboration with several experts in the field and localities throughout the country. This study was carried out by three expert centers for diagnostics and treatment of complex trauma-related psychopathology in refugees and asylum seekers in the Netherlands, namely De Evenaar, Dimence Groep and ARQ Centrum’45. Four MS intervention groups were included in the evaluation. The groups were provided in different languages: Dari, Arabic, Tigrinya, and Ukrainian, which represented the four largest groups of refugees in the Netherlands (Centraal Bureau voor de Statistiek, 2024).

Although every refugee can participate in MS, participants are generally invited by a community officer (e.g., an employment consultant, social worker, or a coordinator at an asylum center). In the present study, localities recruited participants from March to December 2022 in both Dordrecht and Kampen and informed them about the forthcoming study. Upon agreement, the group’s peer educator organized confidential in-person meetings (60–90 min each) in the week before the start of the intervention (T1). No incentives were offered for enrolment in either the intervention or the study. Together with the peer educator, the researcher^2^ explained the study to the prospective participants, obtained their informed consent, recorded their demographic information, and conducted a semi-structured interview about participants’ needs and expectations. Participants then completed standardized quantitative questionnaires, with assistance from the peer educator when needed. As cultural differences may influence the interpretation of constructs such as “resilience” or “sense of coherence,” validated translations of all measures were used whenever available. When no validated version existed, the peer educator provided a translation into the target language prior to administration. The same format was applied in the week following the last session (T3), and participants were asked about their experiences with MS. In addition, the groups’ peer educators administered Cantril’s Ladder at the end of session 2–8 (T2).

The Ethics Committee of Utrecht University, Netherlands, approved the study (number 22–0153).

### 2.4 Measures

#### 2.4.1 Qualitative measure

The qualitative semi-structured interview was designed to complement the quantitative measures and capture participants’ experiences with MS. It was inspired by two items^3^ from the Cultural Formulation Interview (Lewis-Fernández et al., 2016) and aimed to clarify participants’ expectations of MS, as well as to elicit information on their social and life context and any challenges they were facing. Pre-intervention questions focused on housing, access to facilities, work, and social contacts. Post-intervention, participants were asked about their experiences with MS and which aspects they found helpful to their daily lives. An overview of the interview questions is provided in the Appendix.

To ensure the reliability of the qualitative analysis, interviews were systematically coded and categorized using thematic content analysis (Burnard et al., 2008), with independent coding by two authors and consensus discussions to resolve discrepancies. Triangulation with quantitative data further strengthened the robustness of the findings.

#### 2.4.2 Quantitative measures

##### 2.4.2.1 Resilience

The Brief Resilience Scale-6 (BRS-6; Smith et al., 2008) was chosen as the primary outcome and measures a person’s subjective ability to bounce back or recover from stress. Respondents rated six items ranging from ‘Strongly disagree’ (1) to ‘Strongly agree’ (5), on how they perceived their functioning (e.g., *“I tend to bounce back quickly after hard times*.”). The test–retest reliability of the scale among a Saudi-population was good (*r* = 0.88; Baattaiah et al., 2023). In the present study, Cronbach’s Alpha (*α*) was acceptable *α* = 0.696.

##### 2.4.2.2 Well-Being

The WHO-Five Well-Being Index (WHO-5; Topp et al., 2015) measures mental well-being. Participants were asked to rate five positively phrased statements related to the past 2 weeks, varying from ‘Never’ (0) to ‘All the time’ (5). Examples are “*I felt cheerful and in good spirits*” and “*I woke up feeling fresh and rested*.” The reliability of this scale among Syrian refugees in Sweden was good *α* = 0.94 (Tinghög et al., 2017), and acceptable in the present study (*α* = 0.766).

##### 2.4.2.3 Life satisfaction

Life satisfaction was assessed using Cantril’s Ladder, a single-item visual Analog Scale (Cantril, 1966). Participants were asked “*On which step of the ladder do you feel you stand at present.”* The top of the ladder (10) represented the best possible life and the bottom of the ladder (0) the worst. The test–retest reliability of this scale in Kapteyn et al. (2014) was acceptable (*r =* 0.71).

##### 2.4.2.4 Sense of coherence

Sense of Coherence was assessed using Sense of Coherence-Kinderen (SOC-K; Jellesma et al., 2006), a short version of Antonovsky’s Orientation to Life Questionnaire adjusted for adolescents (Torsheim et al., 2001). It measures a dynamic social mechanism consisting of three dimensions: *Comprehensibility, Manageability* and *Meaningfulness*. The construct involves a person’s sense of control over their own life and the extent to which they perceive their life as explicable and predictable. Participants were asked to rate 13 statements such as: *“Do you have the feeling that you are in an unfamiliar situation and do not know what to do?*.” Items ranged from ‘(Hardly) ever’ (1) to ‘(Almost) always’ (5). The reliability of the scale in Super et al. (2018) was good (*α* = 0.83) and acceptable in the present study (*α* = 0.74).

### 2.5 Intervention

The intervention used was MS (Table 1). It is designed as a preventive intervention targeting refugees with emerging or mild psychological complaints rather than those requiring specialized clinical care. The intervention addresses multiple evidence-based components: (i) psychoeducation normalizes stress responses and provides understanding of trauma-related symptoms; (ii) stress management techniques include diaphragmatic breathing and the ‘scale exercise’ for balancing stressful and restorative activities; (iii) cognitive restructuring addresses automatic negative thoughts and teaches reframing techniques; (iv) cultural identity work supports integration strategies based on acculturation theory (Berry, 1997); and (v) peer support through group format and peer educator involvement provides role modeling and social connection. These components align with established therapeutic approaches while being adapted for refugee-specific challenges such as cultural transition, language barriers, and post-migration stressors. The intervention used comprised eight-2 hour weekly- sessions, with 10 to 15 participants. Sessions were delivered by two trainers (a peer educator and a mental health professional) assisted by a registered interpreter. Groups were homogeneous as members had the same cultural and linguistic background. In addition, groups were separated by gender—male and female. The same topics were discussed in each group, though sessions also allowed time to address individual needs of participants.

### 2.6 Data analysis

Descriptive analysis of socio-demographic characteristics at baseline were conducted, i.e., of gender, age, country of origin, juridical status, and time since arrival in the Netherlands.

#### 2.6.1 Evaluation of feasibility

We investigated intervention attendance (i.e., completion, no show, and drop-out) through frequency analysis.

Qualitative data were analyzed using MAXQDA 10 (VERBI) following the four-stage thematic content analysis described by Burnard et al. (2008). Only participants who attended more than four sessions were included. In stage one, the first and last authors (EN and SdlR) independently read all interview minutes and performed open coding of words and short phrases. In stage two, they independently grouped codes into categories, discussing discrepancies until consensus was reached and documenting decisions in detailed coding logs and memos. In stage three, all interview data were organized under these categories, and relevant passages were re-examined to confirm consistency. To further enhance rigor, we reflected on potential researcher biases during regular team discussions and examined findings across language groups to capture cultural differences. It emerged that some findings were particularly relevant in some specific language groups.

#### 2.6.2 Changes in resilience and other mental health domains over time

Changes in resilience and other mental health domains were only evaluated among participants who attended more than half (>4) of the sessions and completed at least two out of four paired measures. All quantitative data analyses were conducted using SPSS 27 (IBM Statistics), considering 95% confidence intervals.

Paired-samples *t*-tests analyzed mean changes from T1 to T3, in resilience, well-being, life satisfaction and sense of coherence. To account for multiple comparisons across the four outcome measures, a Bonferroni correction was applied by adjusting the significance threshold to *α* = 0.0125. *p*-values were evaluated against this corrected threshold to reduce the risk of Type I errors. Cohens’*d* effect sizes were calculated (Cohen, 1988).

Reliable Change Indices (RCI’s) were calculated for each paired measurement. RCI’s assess whether participants’ individual changes on each outcome measure reflect statistically reliable change (i.e., undistorted by measurement error). Unlike *t-*tests, this method provides more information on the variability of intervention responses within a sample and is well-suited for relatively small samples sizes (Jacobson and Truax, 1991). The RCI is the ratio of the difference score and the standard error of the difference score. Individual changes yielding an outcome of ≥ 1.96 or higher and ≤ − 1.96 or lower indicate statistically reliable change, i.e., *actual* improvement or deterioration.

#### 2.6.3 Changes in life satisfaction throughout the intervention

A multilevel analysis (MLA) was applied to test whether life satisfaction, measured repeatedly throughout the intervention, changed over time. This analysis utilizes all available data to estimate the most informed growth curve and is appropriate for samples with missing data, as in the present study (Bickel, 2007). Nevertheless, only participants who attended more than four sessions were included in the analysis. It was built up hierarchically, starting with a null model including only the intercept, then adding the predictor *time* (Model 1; Heck et al., 2014). *Time* denotes the nine time points at which participants’ life satisfaction was assessed. Estimation method Restricted Maximum Likelihood with Satterthwaite degrees of freedom was used.

The intraclass correlation coefficient (ICC) of the null model was calculated to determine the percentage of the variance in life satisfaction attributable to the participant level. A chi-square was calculated based on the model fit indices, −2LL and Bayesian Information Criterion (BIC), to verify whether the model had significantly improved by adding the time predictor. Then, the MLA was repeated, adding *time* as a categorical variable and interpreting the pairwise comparisons. To control for Type I error due to multiple comparisons, a Bonferroni correction was applied to the pairwise tests.

## 3 Results

### 3.1 Participant demographics

Of the *N* = 37 participants 72.37% were female. Their average age was 47.4 years (*SD* = 13.6). The age groups were reasonably equally distributed; 24.32% were aged between 30 and 40 and 29.72% between 40 and 50. On average, participants had lived in the Netherlands for 4.8 years (*SD* = 6.7), though 43% of them had lived in the Netherlands for less than 1 year.

### 3.2 Feasibility

We examined MS’s feasibility through (i) intervention attendance; and (ii) participants’ needs, expectations and experiences with MS.

#### 3.2.1 Intervention attendance

Forty-five (88.24%) of the invited individuals completed the intervention.

#### 3.2.2 Participants’ expectations of and experiences with MS

Thematic content analysis of the semi-structured interviews with participants resulted in several broad themes regarding needs and expectations (pre-intervention), intervention strengths and points for improvement (post-intervention).

##### 3.2.2.1 Pre-MS

Participants stated they wanted to learn about: (i) cultural differences and how to connect with others outside their cultural community; (ii) identity or grief; and (iii) where to find practical support.

##### 3.2.2.2 Post-MS

Participants mentioned the following merits of MS: peer support, the delivery in participants’ mother tongue or their second language and the presence of the peer educator, who was often mentioned as inspiring the group. They also expressed mutual recognition and identification of group members’ challenges and complaints.

*“When you are new in a country, you do not understand anything happening around you which is very stressful. Life becomes easier because of moments like these; you realise: ‘Oh, they are going through it too’.”*

*“Being amongst women only and able to speak our own language gives us the freedom to express ourselves, give input, pose our questions and respond directly to others.”*

[regarding the peer educator] *“It’s really nice to hear from someone who has been here longer than you have, and carries out a message of hope.”*

The following topics were found most helpful: (i) normal responses to stress (psychoeducation); (ii) stress management; (iii) emotion regulation; (iv) cultural differences (and living in a new cultural context), and (v) identity.

*“When I played football, I used to always get angry with myself if I did not do well. MS has taught me how your thoughts affect a situation and that you can change them to something positive.”*

*“I learned that I shouldn’t stay at home all the time. When I sit at home alone, I get tired and sad. Now I go outside and look for distraction and social contact.”*

*“When the language teacher would look at me upon entering the classroom, I used to think: ‘Am I wearing something odd?’ Now I understand its considered polite to make eye contact.”*

*“The theme of identity was very good. It reminded us of our personal skills and talents. Suddenly we felt awakened. Step-by-step we rebuild ourselves.”*

Participants suggested improvement in several area’s. They advised to provide more concrete, practical tools and exercises to manage stress in daily life situations, increase session length and/or frequency and balance practice and talking within the sessions and to organize a 3 month follow-up.

Furthermore it was suggested to offer MS for refugees and asylum seekers immediately upon arrival to all, with a certain degree of obligation. Many participants stated they had wished MS was provided to them much earlier, e.g., shortly after they arrived in the Netherlands.

##### 3.2.2.3 Participants’ experiences per language group; specific needs

The Tigrinya-speaking group mainly consisted of (young) mothers whose biggest challenge was the language barrier and parenting in a new culture. The metaphor of the transplanted tree regrowing its roots was mentioned as helpful to normalize stress responses. They emphasized MS had provided them tools and skills to ask for information and help, e.g., how to sign up for a particular course. Participants wanted more daily distractions/activities. They also stated concerns about their children and suggested refugee youth should be educated on sexual relationships, finances, and addiction.

The Ukrainian-speaking group included women from different regions who had varied war experiences. Participants from eastern Ukraine experienced acute stress, a critical loss of the foundation of their existence, overwhelming emotions, and a need for control. The geographic variations and subsequent differences in exposure to conflict were often mentioned post-intervention. Many participants indicated that this did not consistently help to focus group discussions or enhance the intervention’s relevance for all members. Further, participants stated discussing how to preserve a healthy balance was helpful and suggested fostering active participation through interactive, practical exercises and adding working methods (e.g., yoga, mindfulness or art therapy).

The Dari-speaking group included highly educated women from the municipality and an asylum center. Participants had experienced many losses, e.g., family members, career, and social status. They considered coping with grief, and fostering self-worth to be helpful themes and suggested adding rituals.

The Arabic-speaking men’s group included Yazīdī participants. Many had experienced multiple traumatic events, and several suffered from psychological complaints. There was marked initial distrust toward the researcher and group members, because of their experience with authorities in their home country. Participants often stated their wish to find a job. Helpful themes were: (i) reframing thoughts and perspectives and focusing on personal strengths; and (ii) stress management.

### 3.3 Changes from pre to post-intervention

#### 3.3.1 Improvements in resilience and other mental health domains

Comparisons between pre- and post-intervention assessments on group level found that participants improved on all measured outcomes. Large effect sizes were observed in well-being (*d* = 1.22) and life satisfaction (*d* = 0.9; Table 3). These improvements remained statistically significant after applying a Bonferroni correction.

**Table 3:** Changes in mental health symptoms from pre to post-Mind-Spring in four intervention groups (*n* = 32).

Analyzed pairs	T1		T3		*t*	*df*	*p*	Cohen’s *d*
	*M*	*SD*	*M*	*SD*				
Resilience	16.600	5.017	19.680	3.497	−3.060	24	0.005**	0.612
Well-Being	11.875	5.040	18.000	3.292	−6.892	31	0.001**	1.218
Life Satisfaction	5.844	2.384	8.156	1.417	−5.114	31	0.001**	0.904
Sense of Coherence	40.960	6.522	46.840	5.713	−3.848	24	0.001**	0.770

Mean values (*M*, mean) and standard deviations (*SD*) are reported for each outcome at T1 and T3. Degrees of freedom (*df*) and Cohen’s d effect sizes (0.5 = medium effect, 0.8 = large effect) are displayed for paired-samples t-tests comparing scores at T1 and T3. Statistical significance was determined at *p* < 0.0125, based on a Bonferroni correction for multiple comparisons (four *t*-tests). **p < 0.01 (uncorrected).

#### 3.3.2 Improvements on individual level

RCI’s were calculated for participants who completed a paired measure at T1 and T3. As for resilience, 36% improved (i.e., difference score of ≥ 4.58 points) and 56% remained unchanged and 8% deteriorated. Regarding well-being, 62.5% improved (i.e., difference score of **≥** 3.41 points) and 32.5% remained unchanged. As for life satisfaction 25% improved (i.e., difference score of **≥** 3.52 points), 71.9% remain unchanged and 3.1% deteriorated. Lastly, regarding sense of coherence, 44% improved (i.e., difference score of ≥ 7.74 points) and 56% remained unchanged.

#### 3.3.3 Changes in life satisfaction

Table 4 shows the MLA results of the repeated measurements of Cantril’s Ladder. The (co)variance matrix that best fit the data appeared to be the First Order Autoregressive. The ICC significantly improved when *time* was added in Model 1, χ^2^(1) = 26.16, *p* < 0.05. The estimated coefficient for *time* (*b* = 0.25, *p* < 0.001) indicates that the sample’s mean life satisfaction increased by 0.25 at every sequential time point (i.e., each week).

**Table 4:** Model parameters and goodness of fit for linear changes in life satisfaction.

Effect	Model 0	Model 1
Fixed effects		
	*b* (SE b)	*b* (SE b)
Intercept	6.87** (0.26)	5.95** (0.30)
Time (Level 1)		0.25** (0.04)
Random effects		
First-order autoregressive		
Level 1-variance	3.6	2.72
Level 1-covariance	0.29	0.09
Level 2-intercept	1.79	2.1
ICC	0.33	0.44
Goodness of fit		
-2LL	879.81	853.65
‘BIC	895.78	869.61
Δχ^2^		26.16*
Δdf		1

Standard errors are in parentheses. *p < 0.05. **p < 0.01. In Model 0 the intercept parameter estimate represents the average life satisfaction score based on T1, T2, and T3, across the sample.

**Figure 2:**
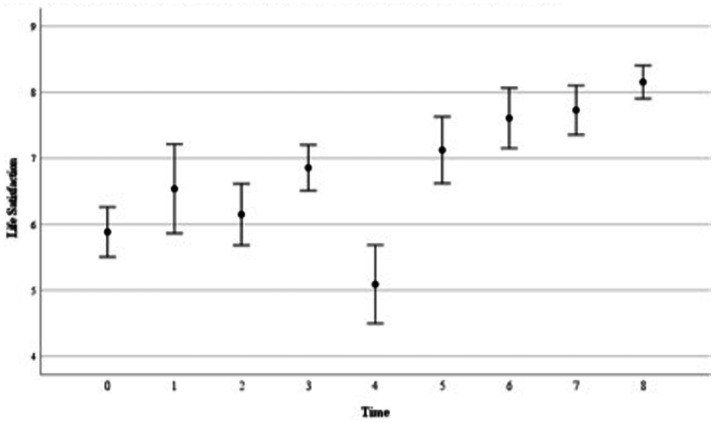
Sample’s mean scores of life satisfaction throughout the intervention. This figure demonstrates the group’s estimated marginal means of the nine life satisfaction assessments, as well as the accompanying standard errors. Time 0 (*n* = 35) represents life satisfaction at pre-intervention (T1). Sessions 2–8 (T2) are indicated at Time 1 (*n* = 13); Time 2 (*n* = 20); Time 3 (*n* = 14), Time 4 (*n* = 11); Time 5 (*n* = 24); Time 6 (*n* = 23) and Time 7 (*n* = 26) respectively, and Time 8 (*n* = 32) signifies life satisfaction post-intervention (T3). It also demonstrates that time significantly predicted participants’ life satisfaction, throughout the intervention.

Estimated marginal means are depicted in Figure 2. Using pairwise comparisons with Bonferroni correction, the average life satisfaction score at Timepoint 0 (*M* = 5.85, *SE* = 0.33) was significantly lower than Timepoint 6 (*M* = 7.71, *SE* = 0.42; *p* = 0.001), Timepoint 7 (*M* = 7.61, *SE* = 0.40; *p* = 0.002) and Timepoint 8 (*M* = 8.08, *SE* = 0.37; *p* = < 0.001). Timepoint 2 (*M* = 6.47, *SE* = 0.45) was lower than Timepoint 8 (*p* = 0.037). Lastly, life satisfaction at Timepoint 4 was lower than Timepoint 6 (*p = 0*.020), Timepoint 7 (*p* = 0.031), Timepoint 8 (*p* = 0.001). These results indicate that participants’ life satisfaction was significantly predicted by *time*. The observed decline at Timepoint 4 appeared statistically insignificant.

## 4 Discussion

The current study indicates that MS may strengthen resilience in a vulnerable group of refugees and asylum seekers from diverse cultural backgrounds. This psychosocial group intervention is culturally tailored and low-threshold, designed to address the unique challenges faced by resettling refugees.

The feasibility and acceptability of MS were found to be high, as indicated by high attendance rates among participants. They valued peer support and a sense of connectedness within the group, consistent with previous research in group interventions (Çetrez et al., 2021; Kananian et al., 2017). They reported feeling understood through both the intervention’s culturally relevant content, which met their needs (Mahon, 2022), and its delivery by a peer educator. This combination of tailored relevant content and delivery by trusted community members is known to foster trust, enhance engagement, and improve the cultural meaningfulness of intervention delivery (de Graaff et al., 2023; Tol et al., 2020; UNHCR, 2024). Group discussions about stress-related complaints fostered mutual recognition and social support. These findings reinforce the importance of culturally sensitive, low-threshold interventions that reduce cultural taboos and stigma surrounding mental health problems (Walther, 2021; Walther et al., 2021).

Group composition and cultural tailoring play a key role in facilitating engagement. The group setting and relative homogeneity, particularly in gender and language, appeared to encourage freer expression among participants, consistent with previous findings (Byström et al., 2023; Hashimoto-Govindasamy and Rose, 2011; Kirsch et al., 2024). Thoughtful group composition can help address gender- and culture-specific coping styles. For example, Çetrez et al. (2021) found that Iraqi refugee women often rely on religious and spiritual coping, whereas men’s use of such strategies may decline post-migration. Literature also suggests tailoring groups to the stage of the refugee journey—e.g., from arrival to legal stabilization—to enhance intervention specificity (Walther, 2021). Yasenok (2025) found significant geographic variation in mental health burdens among Ukrainians, with higher depression, anxiety, and PTSD in south-eastern and central regions—areas more affected by conflict and displacement. This is consistent with our finding that participants from different Ukrainian regions had varied psychosocial needs, highlighting the value of tailoring groups based on region or trauma exposure to enhance cohesion and relevance.

The evaluation of the content of MS showed that several topics were considered to be important: specifically psychoeducation, coping with stress and emotions, cultural differences, and identity. Psychoeducation addressed processes related to stress, trauma, depression, loss, grief, and guilt. It aimed to enhance self-awareness, provide tools, normalize psychological responses, and emphasize the transient nature of these reactions. In post-intervention feedback, participants most valued (i) the “scale exercise,” which involved listing stressful events alongside restorative activities to normalize distress when stressors predominate and highlight the role of pleasurable experiences; (ii) understanding the “vicious circle of stress” of stress, in which recurring thoughts and behaviors perpetuate anxiety and exacerbate rather than resolve problems (Barlow, 2002); and (iii) discussions on automatic thoughts, their emotional impact, and the benefits of reevaluating them to foster a more adaptive outlook (Ellis, 1962). This aligns with Figueiredo and Petravičiūtė (2025) systematic review, which underscores the value of promoting adaptive coping to support psychosocial adjustment and reduce PTSD symptoms in displaced populations, while also stressing the importance of reducing avoidant coping—a maladaptive strategy also addressed in MS.

The findings indicate that MS can be adapted for different groups and tailored to diverse needs, taking into account contextual factors that might influence its effectiveness and feasibility. Proposed improvements included small practical changes and greater emphasis on specific topics, with slight variations across language groups. The Arabic-speaking men’s group emphasized stress management, healthy balance, and personal strengths. They also had work-related questions; a finding also described by Husby et al. (2020). The Tigrinya-speaking group wanted more focus on parenting in a new culture, addiction, and guiding Eritrean youths in handling freedom—similar to findings by van Es et al. (2019) among Eritrean unaccompanied minors in the Netherlands.

Overall, quantitative analyses revealed substantial changes in participants’ outcomes over the course of the intervention. The repeated measures indicated significant improvements in life satisfaction during the intervention. Pre–post measures showed positive effects on all outcomes, with resilience scores increasing from ‘normal’ to ‘high’ levels (Smith et al., 2008). When contextualized within the broader landscape of refugee interventions, these effect sizes are notable. For example, the effect size for well-being was nearly double that reported by Husby et al. (2020) in their study of MS using the same resilience measure, and improvements in life satisfaction were also large. Medium-to-large effect sizes were observed for sense of coherence and resilience. In contrast, an RCT of Group PM + in Turkey reported smaller effects for anxiety, depression, PTSD, and self-identified problems (Acarturk et al., 2022b), while a pilot of 7ROSES in the Netherlands found smaller correlations for general psychopathology and self-efficacy (van Heemstra et al., 2019). However, direct comparison across studies is challenging due to methodological differences, population characteristics, and outcome measures. What our findings suggest is that MS may achieve meaningful improvements across multiple domains, particularly for individuals with mild to moderate psychological distress. At the individual level, approximately half of participants demonstrated positive reliable change on the WHO-5 and SOC-K scales, indicating within-person improvement in well-being and sense of coherence, which has been linked to better mental health among refugees (van Sint Fiet et al., 2022). Although positive individual RCI changes on the BRS and Cantril’s Ladder were slightly lower than for the WHO-5 and SOC-K, MS still produced more favorable distributions than comparable Dutch interventions aimed at strengthening resilience (Hendriks et al., 2024; van Heemstra et al., 2019). Overall, these findings suggest that MS may achieve larger gains across all measured outcomes, alongside meaningful improvements in other psychosocial domains.

It is important to acknowledge that the evidence base for refugee mental health interventions varies in methodological rigor. While randomized controlled trials remain the gold standard, many studies in this field face practical constraints that limit their scope or follow-up duration. Our observational design, while limiting causal inference, offers several methodological strengths including repeated measures analysis, mixed-methods triangulation, and robust statistical approaches with multiple comparison corrections. The retention rates achieved (88.24% intervention completion, 78.72% data provision) compare favorably to other refugee intervention studies, which commonly report dropout rates of 20–30% or higher due to the complex circumstances facing this population. These retention rates themselves provide evidence of MS’s acceptability and feasibility.

While most participants showed improvement, a few did not. One participant with severe complaints pre-intervention demonstrated a deteriorated RCI score post-intervention (Djelantik et al., 2022). These findings suggest that for individuals with severe mental health challenges, MS alone may be insufficient, but it can play an important role in identifying unmet clinical needs and facilitating appropriate referrals. This proposes the intervention is most appropriate for individuals with mild or emerging complaints, and for those facing substantial barriers to accessing mental health services, rather than for those with severe problems requiring specialized care (Baird et al., 2017; Kiselev et al., 2020).

Importantly, this study demonstrates that many participants sought support for practical needs—including administrative tasks, housing, language courses, parenting, and employment. Such post-migration living problems have been found to consistently predict poorer mental health and impaired functional outcomes (e.g., social participation, employment, overall well-being) among refugees (Byrow et al., 2022) and often mediate or exacerbate the impact of pre-migration trauma (Li et al., 2016). In addition, Walther (2021) showed that contextual integration policies and related stressors can undermine refugees’ mental health and integration in Germany. Participant feedback in our study mirrors this broader literature, highlighting that effective embedding of mental health support within practical integration services is crucial. We therefore recommend making MS routinely available within such services to address mental health needs while guiding participants to appropriate practical support. Offering the intervention promptly upon refugees’ arrival, as suggested by participant feedback, could further enhance its relevance and impact.

### 4.1 Limitations and suggestions for future research

While our study offers valuable perspectives and promising findings, certain limitations warrant consideration alongside acknowledgment of the broader methodological challenges in refugee intervention research. The relatively small sample size reduces statistical power, though it is comparable to many studies in this field. The sample’s demographic skew (72.37% female; mean age = 47.4 years) may limit generalizability to younger refugees, males, and other subgroups. Slight differences in group sizes may also have introduced sampling imbalances across cultural groups.

The observational design without a randomized control group limits causal inference; improvements could reflect confounding influences such as time effects, social integration, or other support services. However, the repeated measures and integration of qualitative with quantitative data strengthen our ability to attribute changes to the intervention. Outcomes were measured only immediately post-intervention, precluding durability assessment; future studies should include follow-up assessments to determine whether MS’s effects persist, as has been demonstrated for some other interventions (de Graaff et al., 2024) but not others (Purgato et al., 2021).

The use of self-report measures can introduce potential biases, as well as culturally specific interpretations of mental health constructs. However, our mixed-methods design and the involvement of peer educators—who clarified items during assessment—likely minimized these risks. Future studies could benefit from systematically quantifying feasibility indicators (participant satisfaction, cultural relevance, logistical barriers) to provide a more comprehensive understanding of MS’s applicability in different contexts.

Finally, the Arabic-speaking men’s group included participants from both Syria and Iraq. Grouping refugees from multiple cultural backgrounds may have affected the intervention’s specificity, as coping styles and mental health conceptualizations can vary across cultures (Kirmayer et al., 2011).

## 5 Conclusion

This study provides encouraging evidence that MS is a feasible, acceptable, and culturally meaningful intervention for resettling refugees, and is associated with improvements in key mental health outcomes. Its adaptability, capacity to foster peer support, and potential to identify individuals in need of further care highlight its value as both a preventive and bridging intervention. While the methodological limitations of our observational design must be acknowledged, our findings contribute meaningfully to the growing but methodologically diverse evidence base for culturally adapted psychosocial interventions. MS represents one promising approach among several available interventions, each with their own strengths and limitations. The favorable retention rates and substantial effect sizes suggest MS merits further investigation through randomized controlled trials and could serve as a valuable component within broader, multi-tiered refugee mental health and integration services.

## Data Availability

The raw data supporting the conclusions of this article will be made available by the authors, without undue reservation.

## Footnotes

^1^This number includes 32 million refugees; 6 million Palestinian refugees and 5.8 million other people in need of international protection under the UNHCR’s mandate.

^2^To ensure consistency, the first author of this paper conducted each measurement with the groups’ peer educator.

^3^Items 6 and 7 regard stressors and supports in an individual’s life context from their point of view.

## Appendix

Examples of questions posed during semi-structured interviews.

Example questions pre-intervention:

- Now that you have come to live here in the community, how are you doing? (topics: e.g. housing, facilities, work, social contacts)
- What would you like to learn?
- What are your expectations of the intervention?
- What do you hope will improve in your life, after the intervention?

Example questions post-intervention:

- How do you look back on MS? Which sessions were interesting to you?
- Which skills have you acquired? In which areas do you feel stronger?
- What has improved in your daily life?
